# Supplementary material for: Performance and Safety of EUS Ablation Techniques for Pancreatic Cystic Lesions: A Systematic Review and Meta-Analysis
Source: Cancers (Basel). 2023 May 5;15(9):2627. doi: 10.3390/cancers15092627 (PMC10177078; doi:10.3390/cancers15092627)
Supplement: Supplementary file 1 [file cancers-15-02627-s001.zip › cancers-2362173-supplementary.pdf]

Supplementary Table S1. PRISMA 2020 checklist of the presented objects in this review

Section and Topic	Item #	Checklist item	Reported (Yes/No)
**TITLE**			
Title	1	Identify the report as a systematic review.	YES/p1
**BACKGROUND**			
Objectives	2	Provide an explicit statement of the main objective(s) or question(s) the review addresses.	YES/p1
**METHODS**			
Eligibility criteria	3	Specify the inclusion and exclusion criteria for the review.	YES/p1
Information sources	4	Specify the information sources (e.g. databases, registers) used to identify studies and the date when each was last searched.	YES/p1
Risk of bias	5	Specify the methods used to assess risk of bias in the included studies.	YES/p1
Synthesis of results	6	Specify the methods used to present and synthesise results.	YES/p2
**RESULTS**			
Included studies	7	Give the total number of included studies and participants and summarise relevant characteristics of studies.	YES/p2
Synthesis of results	8	Present results for main outcomes, preferably indicating the number of included studies and participants for each. If meta-analysis was done, report the summary estimate and confidence/credible interval. If comparing groups, indicate the direction of the effect (i.e. which group is favoured).	YES/p2
**DISCUSSION**			
Limitations of evidence	9	Provide a brief summary of the limitations of the evidence included in the review (e.g. study risk of bias, inconsistency and imprecision).	YES/p2
Interpretation	10	Provide a general interpretation of the results and important implications.	YES/p2

Section and Topic	Item #	Checklist item	Location where item is reported
**TITLE**			
Title	1	Identify the report as a systematic review.	Page 1
**ABSTRACT**			
Abstract	2	See the PRISMA 2020 for Abstracts checklist.	Pages 1-2
**INTRODUCTION**			
Rationale	3	Describe the rationale for the review in the context of existing knowledge.	Pages 2-3
Objectives	4	Provide an explicit statement of the objective(s) or question(s) the review addresses.	Page 3
**METHODS**			
Eligibility criteria	5	Specify the inclusion and exclusion criteria for the review and how studies were grouped for the syntheses.	Page 3
Information sources	6	Specify all databases, registers, websites, organisations, reference lists and other sources searched or consulted to identify studies. Specify the date when each source was last searched or consulted.	Page 3-4
Search strategy	7	Present the full search strategies for all databases, registers and websites, including any filters and limits used.	Page 4
Selection process	8	Specify the methods used to decide whether a study met the inclusion criteria of the review, including how many reviewers screened each record and each report retrieved, whether they worked independently, and if applicable, details of automation tools used in the process.	Page 4
Data collection process	9	Specify the methods used to collect data from reports, including how many reviewers collected data from each report, whether they worked independently, any processes for obtaining or confirming data from study investigators, and if applicable, details of automation tools used in the process.	Page 4
Data items	10a	List and define all outcomes for which data were sought. Specify whether all results that were compatible with each outcome domain in each study were sought (e.g. for all measures, time points, analyses), and if not, the methods used to decide which results to collect.	Page 4
	10b	List and define all other variables for which data were sought (e.g. participant and intervention characteristics, funding sources). Describe any assumptions made about any missing or unclear information.	Page 4
Study risk of bias	11	Specify the methods used to assess risk of bias in the included studies, including details of the tool(s)	Page 4

Section and Topic	Item #	Checklist item	Location where item is reported
assessment		used, how many reviewers assessed each study and whether they worked independently, and if applicable, details of automation tools used in the process.	
Effect measures	12	Specify for each outcome the effect measure(s) (e.g. risk ratio, mean difference) used in the synthesis or presentation of results.	Page 4
Synthesis methods	13a	Describe the processes used to decide which studies were eligible for each synthesis (e.g. tabulating the study intervention characteristics and comparing against the planned groups for each synthesis (item #5)).	Page 4
	13b	Describe any methods required to prepare the data for presentation or synthesis, such as handling of missing summary statistics, or data conversions.	Page 4
	13c	Describe any methods used to tabulate or visually display results of individual studies and syntheses.	Page 4
	13d	Describe any methods used to synthesize results and provide a rationale for the choice(s). If meta-analysis was performed, describe the model(s), method(s) to identify the presence and extent of statistical heterogeneity, and software package(s) used.	Page 4
	13e	Describe any methods used to explore possible causes of heterogeneity among study results (e.g. subgroup analysis, meta-regression).	Page 4
	13f	Describe any sensitivity analyses conducted to assess robustness of the synthesized results.	
Reporting bias assessment	14	Describe any methods used to assess risk of bias due to missing results in a synthesis (arising from reporting biases).	Page 4
Certainty assessment	15	Describe any methods used to assess certainty (or confidence) in the body of evidence for an outcome.	
**RESULTS**			
Study selection	16a	Describe the results of the search and selection process, from the number of records identified in the search to the number of studies included in the review, ideally using a flow diagram.	Page 5, Figure 1
	16b	Cite studies that might appear to meet the inclusion criteria, but which were excluded, and explain why they were excluded.	Page 5, Figure 1
Study characteristics	17	Cite each included study and present its characteristics.	Page 5-6, Table 1
Risk of bias in studies	18	Present assessments of risk of bias for each included study.	Page 8 and suppl Table 2
Results of individual studies	19	For all outcomes, present, for each study: (a) summary statistics for each group (where appropriate) and (b) an effect estimate and its precision (e.g. confidence/credible interval), ideally using structured tables or plots.	Pages 8-9
Results of syntheses	20a	For each synthesis, briefly summarise the characteristics and risk of bias among contributing studies.	Page 8-9, suppl table 2
	20b	Present results of all statistical syntheses conducted. If meta-analysis was done, present for each the summary estimate and its precision (e.g. confidence/credible interval) and measures of statistical heterogeneity. If comparing groups, describe the direction of the effect.	Page 8-9, figures 2,3 and suppl fig 1,2
	20c	Present results of all investigations of possible causes of heterogeneity among study results.	Page 8-9 suppl fig 3.4.5
	20d	Present results of all sensitivity analyses conducted to assess the robustness of the synthesized results.	
Reporting biases	21	Present assessments of risk of bias due to missing results (arising from reporting biases) for each synthesis assessed.	Page 9 Suppl fig 6
Certainty of evidence	22	Present assessments of certainty (or confidence) in the body of evidence for each outcome assessed.	
**DISCUSSION**			
Discussion	23a	Provide a general interpretation of the results in the context of other evidence.	Page 10
	23b	Discuss any limitations of the evidence included in the review.	Page 12
	23c	Discuss any limitations of the review processes used.	Page 12
	23d	Discuss implications of the results for practice, policy, and future research.	Page 10-12

Supplementary Table S2. Quality Assessment using the Newcastle-Ottawa Scale (NOS)

Study	Representativeness	Selection	Ascertainment of exposure	Demonstration that outcome of interest was not present at start of study	Factors comparable between groups?	Assessment of outcome?	Follow-up	Adequacy of follow-up	Overall Quality Score (Maximum = 9)
	Population based?	Drawn from same community as exposed cohort	Secured records, clinical outcomes	Not present	Yes? (two points)	Independent blind assessment, record linkage	Mentioned or not mentioned	Complete follow-up, or subjects lost to follow-up unlikely to introduce bias	
Barthet et al.	★	★	★	★	-	★	★	★	7
Caillol et al.	★	★	★	★	-	★	★	★	7
Choi et al. (2017)	★	★	★	★	-	★	★	★	7
Choi et al. (2019)	★	★	★	★	-	★	★	★	7
DeWitt et al.	★	★	★	★	★	★	★	★	9
DiMaio et al.	★	★	★	★	-	★	★	★	7
Du et al.	★	★	★	★	-	★	★	★	7
Gan et al.	★	★	★	★	-	★	★	★	7

Gómez et al.	★	★	★	★	-	★	★	★	**7**
Kim et al.	★	★	★	★	-	★	★	★	**7**
Linghu et al.	★	★	★	★	-	★	★	★	**7**
Moyer et al.	★	★	★	★	★	★	★	★	**9**
Oh et al. (2020)	★	★	★	★	-	★	★	★	**7**
Oh et al. (2011)	★	★	★	★	-	★	★	★	**7**
Park et al.	★	★	★	★	-	★	★	★	**7**

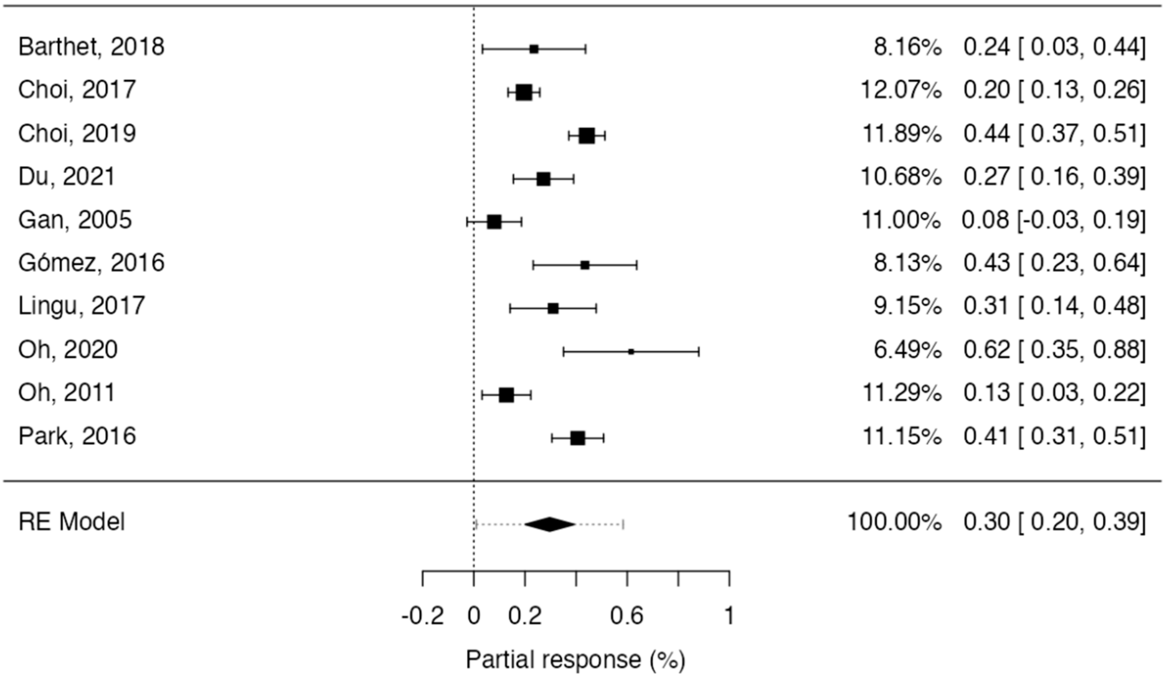

Supplementary Figure S1. Forest plots reporting pooled results of the meta-analysis concerning partial response to EUS-ablation

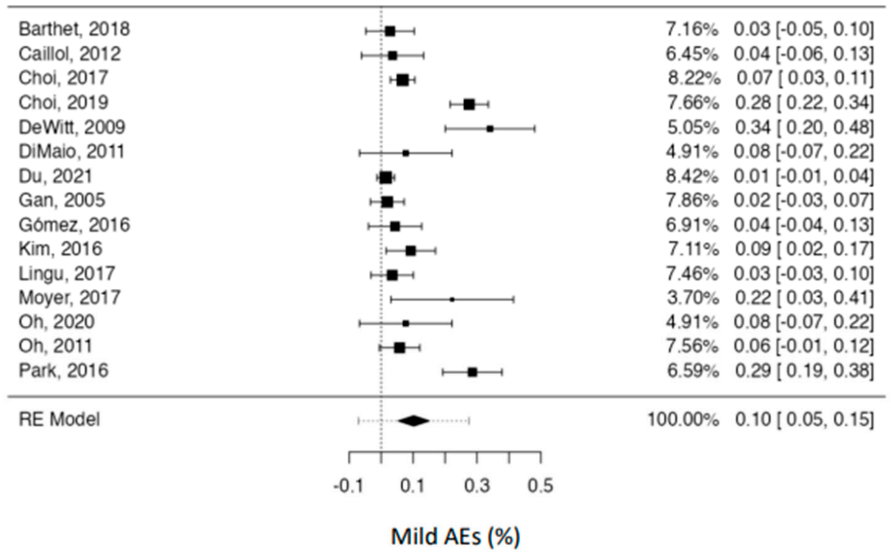

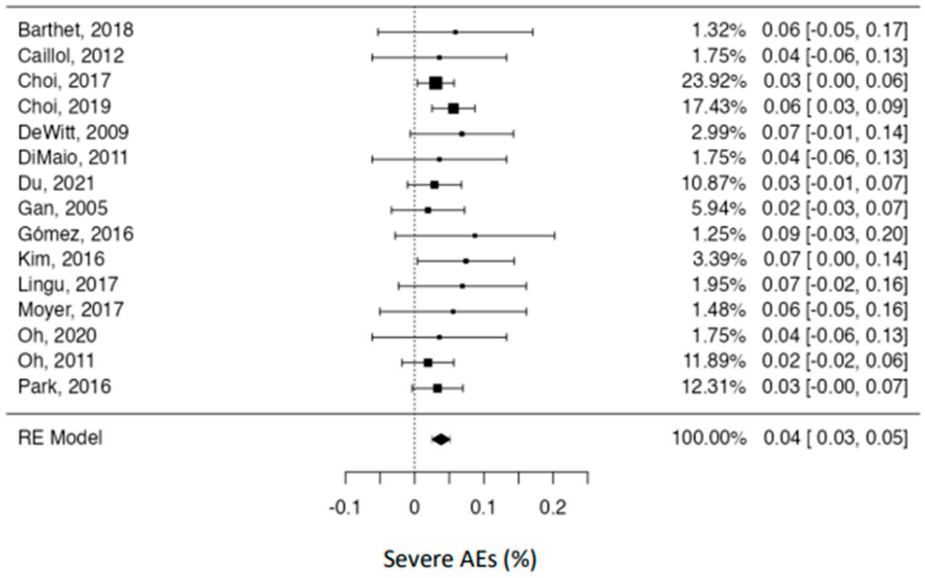

Supplementary Figure S2. Forest plots reporting pooled results of the meta-analysis concerning (a) mild adverse events rate and (b) severe adverse events rate

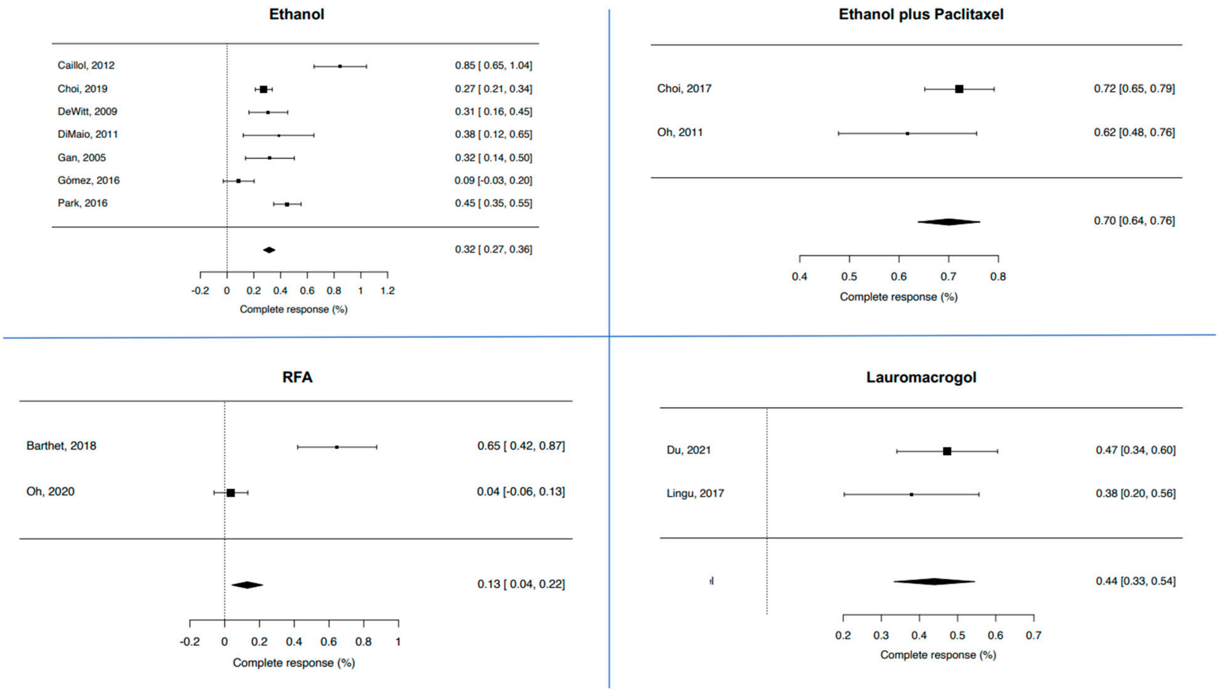

Supplementary Figure S3. Forest plot of the subgroup analysis concerning complete cyst resolution

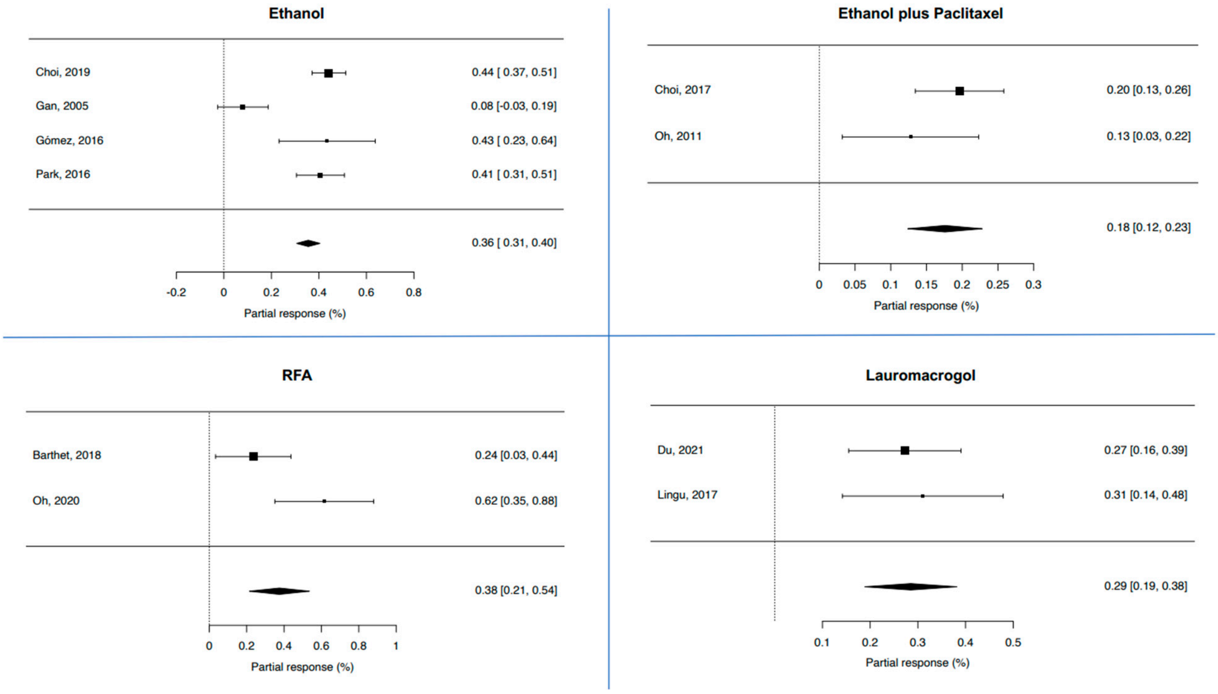

Supplementary Figure S4. Forest plot of the subgroup analysis concerning partial response to EUS-ablation

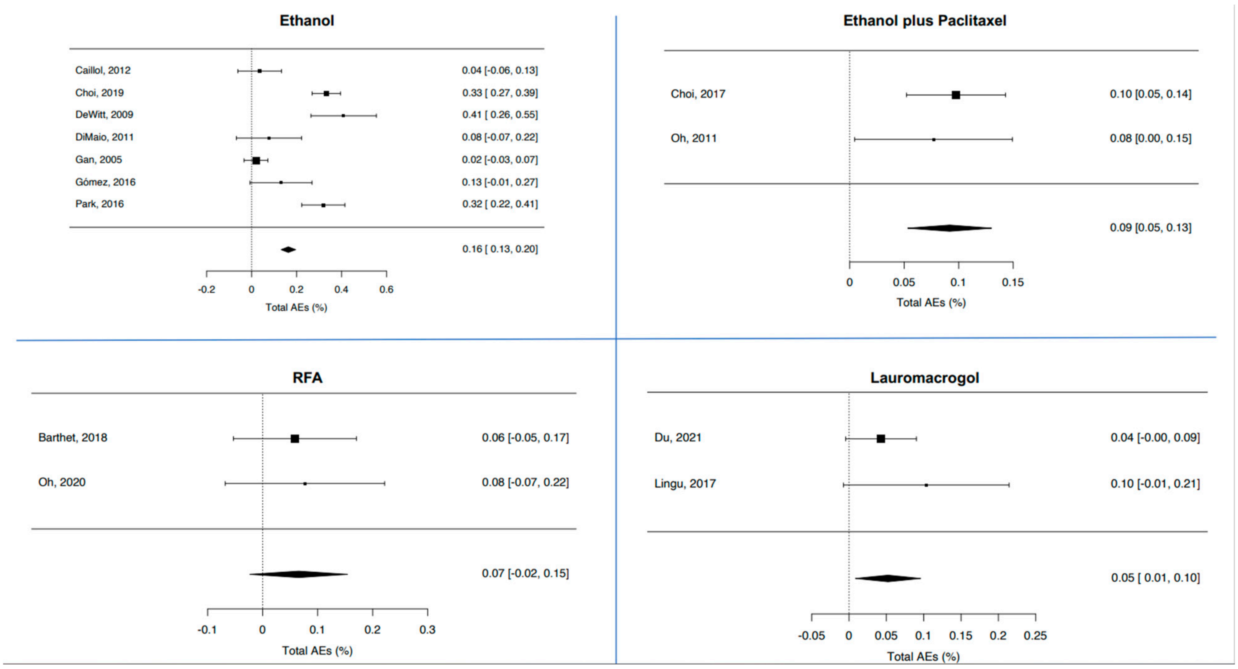

Supplementary Figure S5. Forest plot of the subgroup analysis concerning adverse events rates

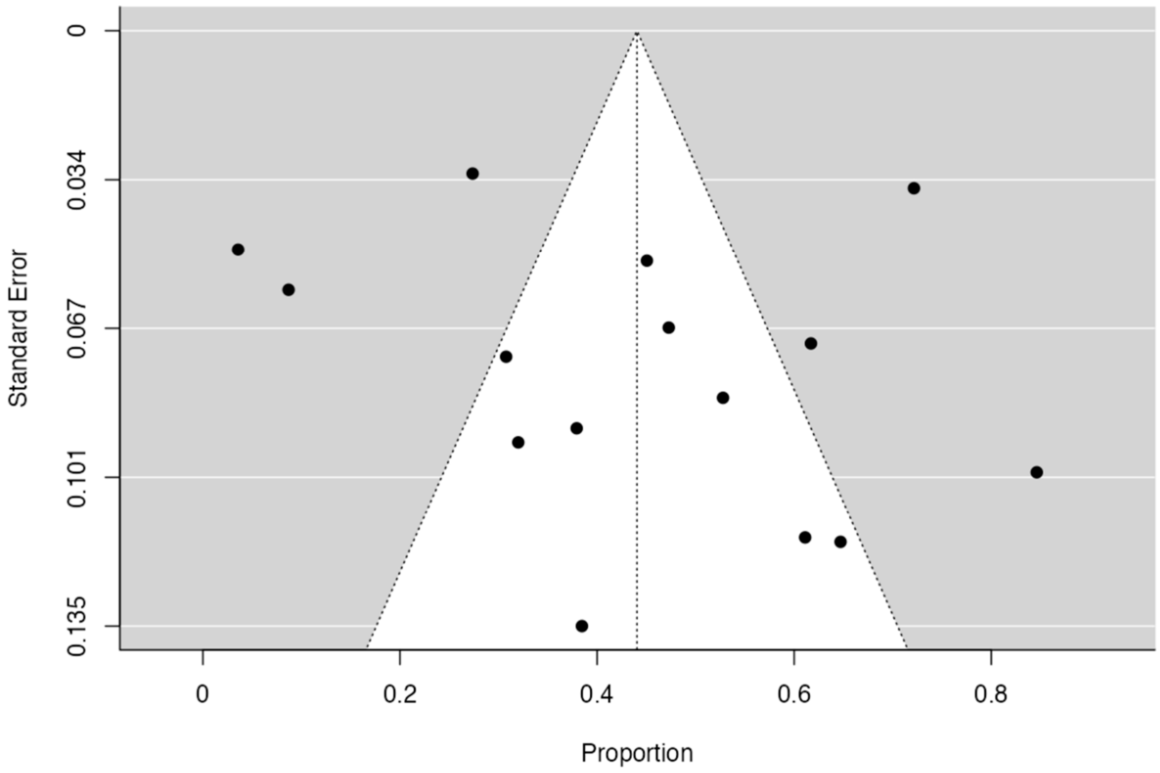

Supplementary Figure S6. Funnel plot reporting publication bias of the analysis concerning the primary outcome of technical success rate.
